# Imaging-Guided Plaque Modification Enables Dual-Branch Revascularization in Unprotected Aorto-Ostial Left Main Chronic Total Occlusion

**DOI:** 10.1016/j.cjco.2026.01.007

**Published:** 2026-01-23

**Authors:** Hidenari Matsumura, Kenichiro Shimoji

**Affiliations:** Department of Cardiology, Saiseikai Utsunomiya Hospital, Tochigi, Japan

**Keywords:** ostial chronic total occlusion, left main, directional coronary atherectomy

Aorto-ostial chronic total occlusion (CTO) of the left main artery (LM) is extremely rare, accounting for ∼0.1% of CTOs.^1^^,^^2^ Most reported cases have involved protected LM-CTO. However, no prior reports have described an unprotected LM-CTO in which the occlusion was extensive enough to involve both ostia completely and no communication existed between the left anterior descending artery (LAD) and left circumflex artery (LCX). We present an unprotected aorto-ostial LM-CTO with a disconnected bifurcation successfully treated using imaging-guided percutaneous coronary intervention (PCI), achieving dual-branch revascularization.

A man aged 70 years with prior left internal thoracic artery–LAD bypass presented with exertional angina. Angiography showed proximal graft occlusion and aorto-ostial LM-CTO (Fig. 1A; Video 1 , view video online). Right coronary injection demonstrated non-interventional epicardial collaterals to both the LAD and the LCX (Fig. 1, B and C; Videos 2  and 3 , view videos online], and computed tomography confirmed bifurcation disconnection (Fig. 1D). After discussion with cardiac surgery, redo coronary artery bypass grafting was considered but deemed unfavourable because the LITA had been used already, redo sternotomy carried a high risk of mediastinal infection, and LCX graft patency was a concern. Therefore, PCI was selected as the preferred strategy for complete revascularization.

**Figure 1:**
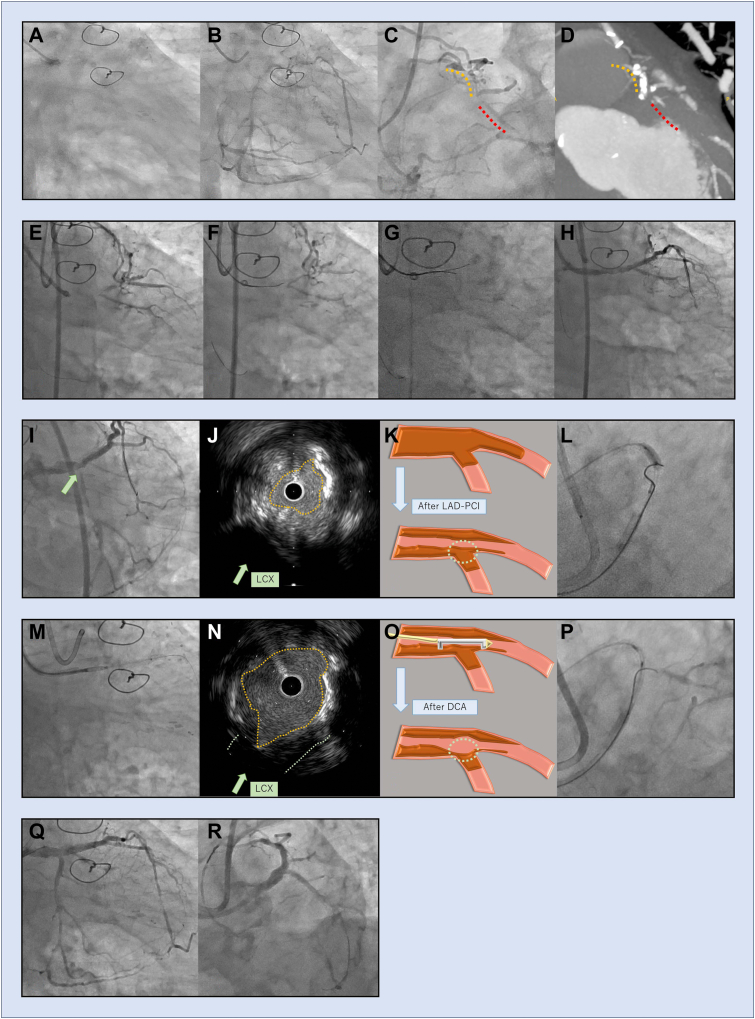
(**A-D**) Pre-coronary angiography and cardiac computed tomography showed left main artery chronic total occlusion involving “disconnected bifurcation” (Videos 1-3, view videos online). (**E-H**) Left anterior descending artery percutaneous coronary intervention (LAD PCI). Initial antegrade wiring with Gaia Next 3 (Asahi Intecc, Nagoya, Japan) failed to penetrate the distal cap. A parallel wire technique using Confianza Pro 12 (Asahi Intecc) successfully crossed the lesion. **(I-L**) After LAD PCI, intravascular ultrasound revealed that the entry was covered by thick plaque, and even Confianza 8-20 (Asahi Intecc) could not penetrate the cap (Video 4, view video online). (**M-P**) After directional coronary atherectomy (DCA; Video 5, view video online), intravascular ultrasound confirmed a reduction of the plaque covering the cap, and the Confianza 8-20 successfully penetrated the cap (Videos 6 and 7, view videos online). (**Q, R**) Final coronary angiography (Videos 8 and 9, view videos online). LCX, left circumflex artery.

PCI was initially performed toward the LAD CTO (Fig. 1E). Initial antegrade wiring with Gaia Next 3 (Asahi Intecc, Nagoya, Japan) failed (Fig. 1F), whereas a parallel wire technique using Confianza Pro 12 (Asahi Intecc) successfully crossed the lesion (Fig. 1, G and H]. After successful LAD crossing, the recanalized left main functioned as the proximal true lumen for the LCX CTO; however, the lumen remained extremely restricted. Moreover, due to the excessive plaque burden, the CTO entry toward the LCX ostium could not be identified by either angiography or intravascular ultrasound at this stage. In CTO PCI, adequate preparation and expansion of the proximal true lumen is essential before aggressive wiring is performed. Accordingly, the LM was dilated with a 4.0-mm cutting balloon. However, intravascular ultrasound revealed that the LCX ostium was still completely covered by thick fibro-calcific plaque (Fig. 1, I-K; Video 4 , view video online). Therefore, after wiring the LAD, we first attempted to puncture the LCX using a dual-lumen microcatheter. However, even with stiff guidewires including Confianza 8-20 (Asahi Intecc) in combination with the balloon-screen technique, proximal cap penetration was unsuccessful (Fig. 1L). Thus, directional coronary atherectomy (DCA, using Atherocut L-9 mm, Nipro Corporation, Osaka, Japan) was performed to remove the plaque and expose the true entry, enabling successful crossing with the Confianza 8-20 (Fig. 1, M-P; Videos 5-7 , view videos online]. Lesion preparation with kissing balloon inflation was followed by mini-crush stenting and final kissing balloon inflation, achieving complete revascularization of both branches (Fig. 1, Q and R; Videos 8  and 9 , view videos online].

Although DCA is approved only in Japan, its fundamental concept of “entry-site plaque modification” also can be achieved with rotational or orbital atherectomy, depending on plaque characteristics and device availability. In particular, when the therapeutic goal is not complete plaque removal, a similar effect may be obtained in fibrocalcific lesions using rotational or orbital atherectomy.^3^ This case highlights that precise imaging and targeted plaque modification can enable safe dual-branch revascularization in unprotected aorto-ostial LM-CTO.

**Novel Teaching Points**

• Unprotected aorto-ostial LM-CTO involving both LAD and LCX ostia with no interbranch communication is extremely rare.
• Intravascular imaging is essential for accurately identifying a thick plaque cap completely covering the side-branch ostium in complex ostial LM lesions.
• When conventional techniques—such as dual-lumen microcatheter wiring or the balloon-screen method—fail to achieve branch penetration, plaque-modifying debulking devices can provide a valuable alternative.
• Even in centres without access to DCA, the underlying concept of “entry-site plaque modification” can be applied using other atherectomy devices.

## Supplementary Material

Video 1 Pre-CAG LCA. CAG of the LCA demonstrating the aorto-ostial LM CTO.

Video 2 Pre-CAG RCA. CAG of RCA demonstrating collateral channels supplying both the LAD and LCX.

Video 3 Pre-CAG RCA (additional view). The occlusion was extensive enough to involve both ostia completely, with no communication between the LAD and LCX.

Video 4 IVUS images after LAD PCI. A thick fibrocalcific plaque is seen completely covering the LCX ostium.

Video 5 DCA to the LM. Directional coronary atherectomy was performed to debulk the plaque covering the LCX ostium.

Video 6 IVUS images after DCA. IVUS demonstrated removal of the thick plaque, with clear exposure of the entry site toward the LCX CTO.

Video 7 CTO puncture with Confianza 8-20. The guidewire is shown engaging and penetrating the CTO entry site.

Video 8 Final-CAG. Successful dual-branch revascularization is demonstrated.

Video 9 Final-CAG (additional view) The bifurcation is well preserved after the procedure. CAG = Coronary angiography, LCA = Left coronary artery, LM = Left main, CTO = Chronic total occlusion, RCA = Right coronary artery, LAD = Left anterior descending artery, LCX = Left circumflex artery, IVUS = intravascular ultrasound, PCI = percutaneous coronary intervention, DCA = directional coronary atherectomy
